# Clinical outcomes in newborns of pregnant women with COVID-19: integrative review

**DOI:** 10.1590/0034-7167-2023-0400

**Published:** 2024-06-28

**Authors:** Iara Bezerra da Silva Ximenes, Cassiano Richel Ferreira Leal, Odinéia Maria Amorim Batista, Maria Eliete Batista Moura, Maria Zélia de Araújo Madeira, Andreia Rodrigues Moura da Costa Valle, Pedro Emílio Gomes Prates, Álvaro Francisco Lopes de Sousa, Denise de Andrade

**Affiliations:** I Universidade Federal do Piauí. Teresina, Piauí, Brazil; II Universidade de São Paulo. Ribeirão Preto, São Paulo, Brazil; III Instituto de Ensino e Pesquisa, Hospital Sírio Libanês. São Paulo, São Paulo, Brazil; IV Nova University of Lisbon. Lisbon, Portugal

**Keywords:** Newborns, Outcomes, COVID-19, Pregnant, Nursing, Recém-Nascidos, Desfechos, COVID-19, Grávidas, Enfermagem, Recién Nacidos, Resultados, COVID-19, Embarazadas, Enfermería

## Abstract

**Objectives:**

to analyze clinical outcomes in newborns of pregnant women with COVID-19.

**Methods:**

integrative review conducted in PubMed, Web of Knowledge, SCOPUS, CINHAL; 2,111 studies were obtained, and 8 articles comprised the final sample.

**Results:**

clinical outcomes in neonates of pregnant women positive for COVID-19 were classified according to the following categories: a) contamination by COVID-19, reported in 62.5% of the studies; b) hospital discharge due to improvement, mentioned in 37.5% of the articles; c) death, representing rare cases in 25% of the sample. The most prevalent gestational complication was prematurity, mentioned in 75% of the studies. This complication has been observed due to cases of premature rupture of membranes and placental abruption.

**Conclusions:**

despite the knowledge of asymptomatic and mildly symptomatic behavior in neonates, it is important to continue the search for new clinical data, as this public has uncertain reactions to SARS-CoV-2 infection.

## RESUMO

**Objetivos:**

analisar desfechos clínicos em recém-nascidos de mulheres grávidas com COVID-19. **M** *étodo* **s:** revisão integrativa realizada no PubMed, *Web of Knowledge*, SCOPUS, CINHAL; foram obtidos 2.111 estudos, e 8 artigos compuseram a amostra final**. Resultados:** os desfechos clínicos em neonatos de mulheres grávidas positivas para COVID-19 foram classificados de acordo com as seguintes categorias: a) contaminação por COVID-19, relatada em 62,5% dos estudos; b) alta hospitalar devido à melhora, mencionada em 37,5% dos artigos; c) óbito, representando casos raros em 25% da amostra. A complicação gestacional mais prevalente foi prematuridade, mencionada em 75% dos estudos. Essa complicação foi observada devido a casos de ruptura prematura de membranas e descolamento de placenta. **Conclus** *ões*: apesar do conhecimento do comportamento assintomático e levemente sintomático em neonatos, é importante continuar a busca por novos dados clínicos, pois esse público apresenta reações incertas à infecção por SARS-CoV-2.

## RESUMEN

**Objetivos:**

analizar los desenlaces clínicos en recién nacidos de mujeres embarazadas con COVID-19.

**Métodos:**

revisión integradora realizada en PubMed, *Web of Knowledge*, SCOPUS, CINHAL; se obtuvieron 2.111 estudios y 8 artículos conformaron la muestra final.

**Resultados:**

los desenlaces clínicos en neonatos de mujeres embarazadas positivas para COVID-19 se clasificaron según las siguientes categorías: a) contaminación por COVID-19, reportada en el 62,5% de los estudios; b) alta hospitalaria debido a mejoría, mencionada en el 37,5% de los artículos; c) fallecimiento, representando casos raros en el 25% de la muestra. La complicación gestacional más prevalente fue la prematuridad, mencionada en el 75% de los estudios. Esta complicación se observó debido a casos de rotura prematura de membranas y desprendimiento de placenta.

**Conclusiones:**

a pesar del conocimiento del comportamiento asintomático y levemente sintomático en los recién nacidos, es importante continuar la búsqueda de nuevos datos clínicos, ya que este público presenta reacciones inciertas a la infección por el SARS-CoV-2.

## INTRODUCTION

At the end of the year 2019, the new Coronavirus (SARS-CoV-2), the virus that causes COVID-19, was identified in the city of Wuhan, China. Due to the progressive increase in cases, rapid spread, and high hospitalization rates, the World Health Organization (WHO) declared the outbreak a pandemic in March 2020. Since then, studies on the mode of transmission, prevention, and treatment were initiated in search of adequate management and control of the disease^(1-2)^.

In Brazil, there was a significant increase in the number of maternal deaths due to COVID-19 comparing the years 2020 and 2021. In the current year, the lethality rate is 7.2%, while the country’s overall lethality rate is 2.8%. Epidemiological Weeks 20 and 21, which took place from May 16 to 29, 2021, showed that in 2020, 544 deaths of pregnant and postpartum women were reported, while by May 2021, there were already 911 confirmed deaths, more frequently observed in pregnant women^(2-4)^.

Physiological and anatomical alterations inherent to gestation may represent an increased risk for diseases with the characteristics of COVID-19, emphasizing the risk of evolution to severe forms of the disease with respiratory decompensation. This is particularly true in the third trimester of gestation, where there may be a need to anticipate delivery. This greater susceptibility characterizes pregnant women as a risk group for SARS-CoV-2 infection^(4-6)^.

SARS-CoV-2 infection in pregnant women can occur at any stage of gestation, with different maternal-fetal impacts depending on the gestational period in which the contamination took place. Newborns (NBs) of pregnant women confirmed for COVID-19 in the third trimester can develop active infection, presenting a risk for adverse outcomes or as potential transmitters for health professionals and caregivers^(5-7)^.

Cases of NBs with SARS-CoV-2 infection are rare, with a predominance of asymptomatic NBs or NBs with mild symptoms. Among the symptoms observed are lethargy, fever, rhinorrhea, cough, tachypnea, dyspnea, nausea, diarrhea, and loss of appetite. However, newborns with secondary and premature diagnostic conditions may show aggravation of the disease^(8)^. Dong et al.^(9)^ demonstrated, in a retrospective study, a greater susceptibility to SARS-CoV-2 in children under one year of age when compared to older ones, a fact probably explained by the immature immune system in neonates^(5)^.

As highlighted by the Brazilian Society of Pediatrics (BSP)^(10)^, according to current scientific knowledge, the routes of transmission of the virus in neonates are droplets and contaminated biological material in the intrapartum and peripartum periods^(8)^. However, the BSP10 does not exclude the possibility of vertical transmission since there are some reports, although rare cases and not non-existent.

Among the maternal-fetal complications related to SARS-CoV-2 infection, the following are observed: premature rupture of the membrane (PROM), premature delivery, fetal distress, and the risk of vertical transmission^(11-12)^. The low amount of data related to the vertical transmission of SARS-CoV-2 prevents knowing its occurrence. However, isolated cases have also become relevant for analysis, such as the identification of IgG and IgM antibodies in neonates born from mothers with COVID-19^(12-14)^.

In this sense, it is observed that despite the rigorous prevention measures for maternal-fetal contamination, the few numbers of studies related to neonatal outcomes prevent the adoption of adequate prevention and control management. This research becomes relevant for this reason, specifically during the pandemic, when many facts remain unknown for this population recognized as vulnerable and more susceptible to contracting infections. This review article also aims to analyze the clinical outcomes in NBs of pregnant women with COVID-19.

## OBJECTIVES

To analyze clinical outcomes in newborns of pregnant women with COVID-19.

## METHODS

### Ethical Aspects of the study

This study followed the ethical principles of research involving human beings established by Resolution 466/2012 of the National Health Council (CNS) and by the Declaration of Helsinki.

### Period, place of study

This is an integrative review. This type of study incorporates scientific evidence, providing a foundation for evidence-based practice, which is so prominent in the fields of health. It is based on the gathering and synthesis of data resulting from research on a topic to be investigated, using systematic and orderly methods in its construction^(15)^.

The elaboration of this review involved the following stages: establishment of the guiding question; identification of inclusion and exclusion criteria; choice of databases to be used; database search and initial analysis of studies for selection; definition of information for categorization of studies; evaluation of studies included in the review; interpretation of results; and presentation of the synthesis of knowledge^(15-16)^.

The guiding question that guided this review was “What are the clinical outcomes in newborns of pregnant women with COVID-19?” structured using the PICo strategy (acronym for Population, phenomenon of Interest and Context). In this regard, it was determined for P: newborns; I: clinical outcomes; Co: COVID-19.

Thus, the proposed search strategy is presented, bearing in mind that in most databases an open strategy was chosen, encompassing all elements of the question. The search date was 10/27/2022. It is noteworthy that, if by chance, combinations of terms did not generate significant or feasible values for the analytical reading, the strategy was expanded or reduced.

Articles were searched in the international biomedical databases PubMed, Web of Knowledge, SCOPUS, Cumulative Index to Nursing and Allied Health Literature (CINHAL). This survey aimed to retrieve the largest possible number of primary studies, combining controlled descriptors (MeSH - Medical Subject Headings terms) and keywords with Boolean operators, as follows:

PubMed: (“neonates”[All Fields] OR “newborns”[All Fields] OR (“infant”[MeSH Terms] OR “infant”[All Fields] OR “infants”[All Fields] OR “infant s”[All Fields])) AND “outcomes”[All Fields] AND (“covid 19”[All Fields] OR “covid 19”[MeSH Terms] OR “covid 19 vaccines”[All Fields] OR “covid 19 vaccines”[MeSH Terms] OR “covid 19 serotherapy”[All Fields] OR “covid 19 serotherapy”[Supplementary Concept] OR “covid 19 nucleic acid testing”[All Fields] OR “covid 19 nucleic acid testing”[MeSH Terms] OR “covid 19 serological testing”[All Fields] OR “covid 19 serological testing”[MeSH Terms] OR “covid 19 testing”[All Fields] OR “covid 19 testing”[MeSH Terms] OR “sars cov 2”[All Fields] OR “sars cov 2”[MeSH Terms] OR “severe acute respiratory syndrome coronavirus 2”[All Fields] OR “ncov”[All Fields] OR “2019 ncov”[All Fields] OR ((“coronavirus”[MeSH Terms] OR “coronavirus”[All Fields] OR “cov”[All Fields]).

Web of knowledge, SCOPUS and CINHAL: Pregnancy AND Perinatal AND newborns AND outcomes AND SARS-COV-2.

### Sample, inclusion and exclusion criteria of the study

The following criteria were included: primary or original articles, published in any language, and without time delimitation, focusing on clinical outcomes of newborns of pregnant women who had a positive RT-PCR for COVID-19 in any gestational trimester or the immediate postpartum period (1 to 10 days after delivery)^(17)^; it was also considered that newborns are individuals aged less than 28 days^(18)^. The following were excluded: review studies, non-epidemiological studies/based on case reports or single cases, expert opinions, protocols, response letters, and editorials; and research that focused on maternal outcomes after COVID-19 infection.

The bibliographic search took place simultaneously in the four databases by two researchers with expertise in the method and subject studied, in different locations, to avoid bias in the screening of articles to be analyzed. Discursive meetings were held aiming at consensus among researchers about the inclusion or exclusion of pre-selected studies. In cases of disagreements that could not be resolved by consensus, a third reviewer was contacted. Review searches, expert opinions, protocols, response letters, and editorials were excluded in the first search. Filtering for the selection of research was carried out in three phases, namely:

I. The articles identified in the databases were pre-selected according to the inclusion criteria and analyzed by reading their titles and abstracts. Thus, each database presented the following number of retrieved studies: PubMed - 995, Web of Knowledge - 572, SCOPUS - 270, CINAHL - 667, resulting in a total of 2,504 primary studies.

II. At this stage, 192 duplicate articles were removed, and the pre-selected research were analyzed regarding the potential for participation, evaluating the fulfillment of the research question, the type of investigation developed, objectives, materials and methods, main results, and conclusion. Thus, the following were excluded: research carried out with the wrong population, with the wrong phenomenon or intervention, with the wrong context, the wrong type of study, which did not allow search and extraction, not available in electronic media, with the wrong type of document (Chart 1).

**Chart 1:** Distribution of justifications with quantitative correspondents of excluded articles

Reason for exclusion	Quantitative excluded (n=2301)
Wrong population	981
Wrong phenomenon or wrong intervention	711
Wrong context	485
Wrong type of study	102
Search and extraction not allowed	10
Not available in electronic format	12

III. In the last phase, the 11 primary studies included were read in full, to collect data specific to the objectives of the review. From these studies, the following were evaluated: bibliometric questions (year, country of publication), methodological design, neonatal outcomes, and possible symptoms presented and related factors.

Integrative review supported or guided by the PRISMA tool. The process review considered the recommendations of the *Preferred Reporting Items for Systematic Reviews and Meta-Analyses* (PRISMA) checklist. The presentation of the discussion and interpretation will be carried out in a descriptive way and from the emergence of data.

**Figure 1:**
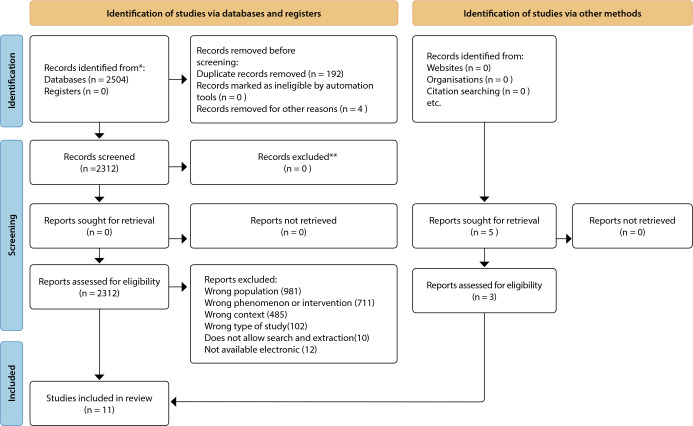
PRISMA flowchart adapted for the search, 2022

The selection flowchart of the 11 articles included in this review and the number of articles excluded with respective justification can be seen in (Figure 1).

## RESULTS

The 11 studies included in the review were all in English (100%). Regarding the year of publication, 2020 was the most prevalent (87.5%) with only one study in 2021. The country that published the most was China, with four (50%) studies that met the inclusion criteria. As for the methodological design, cohort studies were more present, representing 62.5% of the selected articles (Chart 2). In general, the articles included investigated the clinical manifestations of pregnant and postpartum women with COVID-19, and clinical findings in the children of these women.

**Chart 2:** Characterization and main results found in the selected manuscripts

Title	Year/ country	Design/ Participants	Outcomes
Clinical analysis of 10 neonates born to mothers with 2019-nCoV pneumonia^(19)^	2020 China	Retrospective study 10 neonates	Perinatal COVID-19 infection can harm newborns, leading to fetal distress, prematurity, respiratory issues, thrombocytopenia, and death. No evidence of vertical transmission was found.
Clinical characteristics of 19 neonates born to mothers with COVID-19^(20)^	2020 China	Cohort study n = 19 neonates	Neonates showed no clinical, radiological, or hematological evidence of COVID-19, and no vertical transmission occurred during the third trimester.
Maternal and neonatal outcomes of pregnant women with COVID-19 pneumonia: a case-control study^(21)^	2020 China	Cohort study n = 17 neonates	Newborns did not contract COVID-19 or experience serious complications or respiratory failure during hospitalization.
Neonatal Early-Onset Infection with SARS-CoV-2 in 33 Neonates Born to Mothers with COVID-19 in Wuhan, China^(22)^	2020 China	Cohort study n = 33 neonates	Neonatal symptoms in COVID-19-exposed or at-risk infants were mild, with favorable outcomes. Vertical transmission couldn’t be ruled out despite negative test results.
Vaginal delivery in SARS-CoV-2 infected pregnant women in Northern Italy: a retrospective analysis^(23)^	2020 Italy	Retrospective multicenter study n = 42 neonates	Some neonates tested positive for COVID-19, possibly due to postpartum contamination, suggesting low intrapartum transmission risk during vaginal delivery.
Clinical profile, viral load, management and outcome of neonates born to COVID 19 positive mothers: a tertiary care centre experience from India^(24)^	2020 India	Cohort study n = 65 neonates	Seven out of 65 tested neonates had mild COVID-19, and maternal viral load did not correlate with newborn severity.
A multicenter study on epidemiological and clinical characteristics of 125 newborns born to women infected with COVID-19 by Turkish Neonatal Society^(25)^	2020 Turkey	Multicenter cohort n = 125 neonates	Four out of 120 newborns tested positive for COVID-19, indicating significant impacts on perinatal and neonatal outcomes, including prematurity, cesarean section, suspected vertical transmission, and low breastfeeding rates.
Clinical outcomes of maternal and neonate with COVID-19 infection-Multicenter study in Saudi Arabia^(26)^	2021 Saudi Arabia	Retrospective cohort study n = 200 neonates	Most neonates had normal laboratory results, with few requiring NICU admission or respiratory support, and no cases of COVID-19 or evidence of vertical transmission.
Clinical outcomes of COVID-19 positive mothers and their newborns - a retrospective study^(27)^	2022 Portugal	Retrospective cohort study n = 52 neonates	The study covered 865 deliveries, with few infants testing positive for SARS-CoV-2, and no fatal outcomes were observed, indicating unlikely perinatal or postnatal transmission.
Clinical outcomes and antibody transfer in a cohort of infants with in utero or perinatal exposure to SARS-CoV-2 (Coronascope Study)^(28)^	Espanha 2022	Observational prospective study n = 95 neonates	Infants born to mothers with COVID-19 had normal neurological and auditory development, and most exhibited typical development in their first year of life, with only some showing positive serological results.
Shortand mid-term multidisciplinary outcomes of newborns exposed to SARS-CoV-2 in utero or during the perinatal period: preliminary findings^(29)^	Italy, 2022	Prospective study N: 199 newborns.	The study encompassed various assessments, most newborns had normal growth and neurological development, and some showed potential effects of SARS-CoV-2 exposure. Overall, positive results were observed, except for some ophthalmological findings in a subset of cases

The clinical outcomes observed in newborns of pregnant women positive for COVID-19 varied across the analyzed studies. These outcomes were classified into three categories:

- COVID-19 Contamination: This category was reported in the majority of studies, accounting for 62.5% of the analyzed articles.
- Hospital Discharge Due to Improvement: This outcome, indicating successful recovery, was mentioned in 37.5% of the articles.
- Death: While representing a small portion, sparse cases of neonatal death were found in 25% of the sample.

Among the neonatal clinical manifestations observed in these cohorts, the most common were dyspnea (62.5%), 5th-minute Apgar scores below 8 (37.5%), and less frequently reported conditions such as respiratory distress syndrome, fever, and emesis (25%).

It’s noteworthy that although neonatal clinical findings were observed in 75% of the studies, only 50% of the articles documented cases of positive COVID-19 test results. In the 50% of articles where all individuals in the cohort tested negative for SARS-CoV-2 via RT-PCR, evidence of vertical transmission was excluded.

This comprehensive analysis of studies from various countries provides valuable insights into the clinical outcomes of newborns born to COVID-19-positive mothers, shedding light on the multifaceted impact of maternal infection on neonatal health and the potential risk of transmission during the perinatal period.

## DISCUSSION

The studies included in this analysis provide a comprehensive view of the clinical outcomes in newborns of women with COVID-19 during pregnancy. Although some studies^(19-24,27-29)^ have a limited number of samples, they still offer valuable insights into the landscape of neonatal outcomes in this scenario. It is important to note that, despite not being entirely representative, studies like those by Oncel et al.^(25)^ and Al-Matary et al.^(26)^ play a crucial role as they highlight the scarcity of literature focusing on the outcomes of newborns born to mothers with COVID-19 during pregnancy. One possible explanation for this gap is the predominance of asymptomatic and mild symptomatic cases in infants with COVID-19 infection, which may have reduced interest in exploring other outcomes.

All participants in the binomial that made up groups of confirmed cases for COVID-19 underwent RT-PCR test, in a pharyngeal^(22-26)^ or nasopharyngeal^(19-21)^, and anal swab sample^(22)^. Other samples were tested by Liu et al.^(20)^ and Oncel et al.^(25)^, such as: deep tracheal aspirate, breast milk, blood, serum, feces, placental tissue, amniotic fluid, and umbilical cord blood. Li et al.^(21)^ and Zhu et al.^(19)^ also performed computed tomography (CT) of the chest, verifying as alterations, diffuse and/or bilateral ground-glass opacity, irregular lung consolidation and blurred edges. Furthermore, this test, associated with RT-PCR, is part of the clinical diagnostic criteria in the country of origin of these studies^(21)^.

Ferrazzi et al.^(23)^ and Anand et al.^(24)^ describes cases of NBs testing positive for COVID-19 on the 3^rd^ and 4^th^ postpartum days, respectively. In the first clinical finding mentioned above, the neonate was separated from the mother after delivery, developing symptoms a few hours later, and presenting an ambiguous test result, which was positive only after the third day of life. In the cases observed by Anand et al.^(24)^, the newborns had started breastfeeding after delivery, without using masks. However, mothers developed symptoms on the fourth postpartum day, as a result, newborns and postpartum women showed confirmation of testing for the virus.

Among the gestational complications observed in seven of the analyzed studies^(19,21-26)^, the most prevalent was prematurity, being cited in 75% of all studies^(19,21-26)^. Li et al.^(21)^ highlights this complication because of cases of premature rupture of membranes (PRM) and placental abruption (PA), however, Zeng et al.^(22)^ and Anand et al.^(24)^ observed it to be preceded by fetal distress. Furthermore, occurrences of spontaneous prematurity could also be noticed in the cohort of Oncel et al.^(25)^ and Ferrazzi et al.^(23)^. The findings by Ferrazzi et al.^(23)^ show that pregnant women who experienced worsening symptoms of COVID-19 infection, and required cesarean section, had higher rates of premature newborns and low birth weight.

As for the mode of delivery, Ferrazzi et al.^(23)^ emphasize that vaginal delivery is not contraindicated for pregnant women who are positive for COVID-19, since this route does not represent a greater risk of intrapartum contamination of the newborn. However, in cases of worsening maternal symptoms, with or without signs of fetal distress, cesarean section is considered the safest route to the binomial^(23-25)^. On the other hand, in the study sites by Li et al.^(21)^ and Zeng et al.^(22)^, cesarean section was used as standard management for confirmed and suspected cases of maternal pneumonia caused by COVID-19. Other studies did not explain whether the infection influenced the choice of mode of delivery, with both means occurring similarly^(19-20,26)^.

In the investigation by Liu et al.^(20)^, the mother-infant dyad was separated soon after delivery following the standard protocol, for isolating newborns from mothers with the infection. This was like the management used in the field of data collection by Oncel et al.^(25)^, in which neonates were kept in the Neonatal Intensive Care Unit (NICU) if they presented high-risk gestational factors; however, those who did not present such factors were kept at two meters from the mother. In other analyses, the separation of the binomial was only considered in the presence of complications, such as severe maternal or neonatal symptoms^(22-26)^.

The need for respiratory support for newborns was reported in six studies, especially non-invasive support^(19,22-26)^. Zhu et al.^(19)^, Ferrazzi et al.^(23)^ and Anand et al.^(24)^ report that this deficiency was remedied after oxygen therapy for 48 hours, 18 hours and 24 hours, respectively, with hospital discharges for improvement. Li et al.^(21)^ denies the occurrence of cases of respiratory failure.

Several studies did not find evidence of vertical transmission of the virus from mother to newborn. However, limitations in testing specific antibodies (IgM and IgG) in neonatal samples may have affected the validity of these results^(19-21,23-29)^.

This study offers several important implications for nursing practice in maternal-child care and in other healthcare settings^(30-40)^, both in Brazil and worldwide. Nurses working in maternal-child care play a critical role in managing pregnant women with COVID-19 and caring for newborns affected by the infection^(30-31,34-38)^. Adaptability, continuous updating, and an understanding of clinical nuances are essential for providing quality care, both in Brazil and globally, as we face the challenges posed by the COVID-19 pandemic and the post-pandemic era^(32-38)^.

Nurses need to be well aware of potential neonatal outcomes in cases where pregnant women contract COVID-19 and use the lessons learned to apply them to other populations. Understanding the prevalence of different outcomes is vital for nursing professionals, empowering them to provide the necessary care and support to populations^(32-40)^.

### Study limitations

Only 11 articles were included in the final sample obtained from the databases. The relatively small number of studies may be associated with a greater focus on vaccination-related research, which could limit the generalizability of the findings and fail to encompass the full range of clinical outcomes in newborns of pregnant women with COVID-19. Future research should strive to incorporate a larger number of studies to achieve a more comprehensive understanding of this subject.

Additionally, the majority of the included studies were conducted in China, with four out of 11 studies originating from there, implying a geographical bias. Furthermore, all the articles included in the review were in English. This geographical and language bias may limit the applicability of the results to other regions and populations. It is important for future studies to include a more diverse range of geographical locations and languages to ensure a broader representation of clinical outcomes in newborns of pregnant women with COVID-19.

### Contributions to Nursing Area

This study makes significant contributions to the nursing field through its comprehensive synthesis and analysis of literature from diverse sources. By shedding light on various critical outcomes, including rates of COVID-19 infection, patient recovery leading to hospital discharge, and even rare instances of mortality, it equips healthcare professionals, including nurses, with valuable insights for the effective care of newborns affected by COVID-19.

One pivotal finding from this research is the recognition of prematurity as the most prevalent gestational complication observed in newborns born to pregnant women with COVID-19. It further underscores that premature births can be attributed to complications like premature rupture of membranes (PRM) and placental abruption (PA). This information holds immense significance for nurses working in obstetric and neonatal settings, emphasizing the vital role they play in closely monitoring and managing pregnant women with COVID-19. This proactive approach can help prevent adverse outcomes in newborns and ensure the well-being of both mothers and infants during these challenging times.

## CONCLUSIONS

Vertical transmission of the SARS-CoV-2 virus is still an underexplored field of knowledge since there is still no evidence of its occurrence or impossibility. In this sense, further studies on the subject, with the search for specific IgM and IgG antibodies in newborns of mothers with positive tests, have much to add to evidence-based practice.

The clinical outcomes of these NBs referred to in the studies of this review not only involve hospital discharge due to the absence of symptoms, but also a significant number of contaminations by COVID-19 and few cases of deaths. Thus, even with the knowledge of asymptomatic and mild symptomatic behavior in neonates, it is important to continue the search for new clinical data, as this public still has uncertain reactions related to infection by the SARS-CoV-2 virus.

It is added that the use of masks throughout the hospital stay for both positive and negative cases of COVID-19 infection is an essential management measure for the control and prevention of new infections, especially to protect newborns. Maintaining the coordination of these security measures, in addition to performing RT-PCR tests not only at admission, are important means of protecting the spread of the virus, even after vaccination.
